# A mechanistic model for individualised treatment of anxiety disorders based on predictive neural biomarkers

**DOI:** 10.1017/S0033291720000410

**Published:** 2020-04

**Authors:** Anne-Kathrin Brehl, Nils Kohn, Aart Herman Schene, Guillen Fernández

**Affiliations:** 1 Radboud University, Donders Institute for Brain Cognition and Behaviour, Nijmegen, The Netherlands; 2 Radboud University Medical Center, Nijmegen, The Netherlands

**Keywords:** Anxiety, PTSD, social phobia, panic disorder, generalized anxiety disorder, noradrenaline, locus coeruleus, PFC, amygdala, exposure therapy

## Abstract

Increased amygdala responsiveness is the hallmark of fear and a characteristic across patients with anxiety disorders. The amygdala is embedded in a complex regulatory circuit. Multiple different mechanisms may elevate amygdala responsiveness and lead to the occurrence of an anxiety disorder. While top-down control by the prefrontal cortex (PFC) downregulates amygdala responses, the locus coeruleus (LC) drives up amygdala activation via noradrenergic projections. This indicates that the same fearful phenotype may result from different neural mechanisms. We propose a mechanistic model that defines three different neural biomarkers causing amygdala hyper-responsiveness in patients with anxiety disorders: (a) inherent amygdala hypersensitivity, (b) low prefrontal control and (c) high LC drive. First-line treatment for anxiety disorders is exposure-based cognitive behavioural therapy, which strengthens PFC recruitment during emotion regulation and thus targets low-prefrontal control. A treatment response rate around 50% (Loerinc et al., 2015, *Clinical Psychological Reviews*, **42**, 72–82) might indicate heterogeneity of underlying neurobiological mechanisms among patients, presumably leading to high variation in treatment benefit. Transforming insights from cognitive neuroscience into applicable clinical heuristics to categorise patients based on their underlying biomarker may support individualised treatment selection in psychiatry. We review literature on the three anxiety-related mechanisms and present a mechanistic model that may serve as a rational for pathology-based diagnostic and biomarker-guided treatment selection in psychiatry.

## Introduction

Anxiety disorders are among the most common mental health problems. In the United States for instance, the lifetime prevalence to suffer from any anxiety disorder is 33.7% (Kessler, Petukhova, Sampson, Zaslavsky, & Wittchen, 2012). Unlike relatively mild and transient fear caused by threatening events or places, anxiety disorders are characterised by severe and persistent symptoms causing significant impairment in daily life functioning (Mendlowicz, 2000). If untreated, anxiety disorders tend to become chronic conditions (Fifer et al., 1994).

In clinical practice, diagnosis of an anxiety disorder is based on clusters of symptoms rather than the underlying pathophysiology and potential individual neurobiological variation. First choice treatment for anxiety disorders is exposure-based cognitive behavioural therapy (CBT) (Bandelow et al., 2014; Courtois et al., 2016; National Collaborating Centre for Mental Health, 2013). However, the response rate of patients with various anxiety disorders for this initial treatment is in a range of 45% to 65% (Bandelow et al., 2014; Loerinc et al., 2015). There are certainly many reasons why treatment is non-effective in a substantial number of patients. Yet, heterogeneity of underlying neurobiological mechanisms among patients might cause substantial variation in treatment effects. Scientific understanding of underlying individual differences leading to a heterogeneous therapy outcome is still limited. To increase the treatment response, we may be in need of such a more personalised approach. So far, psychiatry still follows a symptom-based approach to classify patients. Moreover, research on neurobiological mechanisms of mental disorders has mainly focused on neural processes common to groups of affected individuals. A shift towards probing individual differences in pathology related neural processes is emerging.

We need models to refine our understanding of the underlying working mechanisms of biomarkers in order to individualise treatment in psychiatry by adapting treatment to individual biological predispositions. Here we propose a neurobiology-based mechanistic model that might help to clarify some of the clinical heterogeneity among patients with anxiety disorders. The model may foster treatment selection tailored to the anxiety evoking neurobiological mechanism of the individual patient.

Research demonstrated that anxiety is characterised by functional and structural alterations in cortical and subcortical areas that have been primarily related to amygdala hyperactivity (Etkin & Wager, 2007). We propose three potential mechanisms that all result in increased amygdala responsiveness: (a) inherent amygdala hypersensitivity, (b) low prefrontal control and (c) high locus coeruleus (LC) drive. There is ample evidence that local, for instance GABAergic mechanisms in the amygdala are critical for amygdala responsiveness (Braga, Aroniadou-Anderjaska, Manion, Hough, & Li, 2004). However, a considerable body of animal and patient-related research points towards noradrenergic projections that drive amygdala hyper-responsiveness. Located in the brain stem, the LC is the major noradrenaline source of the brain (Szabadi, 2013). During acute stress, noradrenaline is released by LC neurons and from there projected to almost the entire brain. Such projections to the amygdala enhance amygdala responsiveness (Hermans et al., 2011), while high noradrenaline release to the prefrontal cortex (PFC) distracts attentional processes and working memory (Arnsten & Li, 2005; Sara, 2009). In contrast, inhibitory prefrontal regulation reduces amygdala responsiveness (Etkin & Wager, 2007; Etkin, Büchel, & Gross, 2015; Kohn et al., 2014). Correspondingly, anxiety disorders have been associated with impaired prefrontal control (Etkin & Wager, 2007; Sylvester et al., 2012).

Taken together, amygdala hyper-responsiveness is a neurobiological correlate of symptoms in anxiety disorders that results from imbalances in amygdala-centred regulatory mechanisms, where the PFC and LC are the core regulatory hubs. Alterations in one of those hubs lead to increased amygdala responsiveness and thus symptoms of anxiety. Adapting treatment specifically to the altered regulatory mechanism might increase therapeutic effects. Since CBT has been shown to increase prefrontal control, patients with reduced prefrontal control may benefit most from CBT (Arnsten, Raskind, Taylor, & Connor, 2015). Amygdala hyper-responsiveness without indication for reduced prefrontal control might be treated more efficiently with pharmacological options, like GABAergic drugs to downregulate intrinsic amygdala hypersensitivity, and *α*2 agonistic drugs targeting the noradrenergic system may reduce LC drive most effectively. Pharmacological studies in post-traumatic stress disorder (PTSD) revealed successful symptom reduction after regulating noradrenaline imbalances with *α*1-receptor antagonists or *α*2A-receptor agonists (Arnsten et al., 2015; Detweiler et al., 2016). As a guideline for potential treatment selection we outline a model that supports treatment selection based on the patient's underlying anxiety evoking neurobiological mechanism. Such a relatively simple and highly pragmatic mechanistic model would allow to allocate patients based on their neurobiological disposition to the most beneficial, personalised treatment. In the first part we lay out the three potential biomarkers of anxiety. In the second part we translate findings from cognitive and clinical neuroscience into biomarker-based treatment selection hypotheses for clinical practice.

## Biomarkers of anxiety

### Amygdala hypersensitivity – mechanism or symptom of anxiety?

Fear is a basic survival mechanism occurring in response to threat. It goes along with a well-orchestrated brain state allowing for appropriate adaptive behaviour. The amygdala is the central structure organising this brain state (Bouret & Sara, 2005; LeDoux, 2003). Such an adaptive fear response is associated with variation in amygdala activation (Phan et al., 2004) and based on the cognitive appraisal of potential threat. The basolateral amygdala receives information about the outside world from the thalamus, the hippocampus and frontal cortex (Davis & Whalen, 2001). In order to adapt behavioural responses adequately, information regarding positive or negative valence of sensory stimuli is (re)transmitted from the basolateral amygdala to diverse cortical regions, especially the midline and orbital prefrontal cortices, for further processing (Janak & Tye, 2015).

The excessive and context inadequate fear response is the main characteristic of anxiety disorders. In humans, the basolateral amygdala has been shown to initiate active escape due to threat (Terburg et al., 2018). Imaging studies demonstrated increased amygdala and insula activity as a shared neuronal response to aversive stimuli across different anxiety disorders (Del Casale et al., 2012; Etkin & Wager, 2007; Fonzo et al., 2010, 2015; Kraus et al., 2018; Linares et al., 2012; Shin & Liberzon, 2009).

On a neurobiological level, glutamate is the major excitatory neurotransmitter in the amygdala (Pape & Pare, 2010). Noradrenaline and GABA are neuro-modulators that affect this excitatory information transfer. Inhibitory GABA-interneurons gate the information flow between the basolateral amygdala and the central amygdala (Royer, Martina, & Paré, 1999) and counterbalance excitatory action of glutamate (Lydiard, 2003), while noradrenaline suppresses GABAergic inhibition (Tully, Li, Tsvetkov, & Bolshakov, 2007). Alterations in those modulators, like decreased GABA transmission and increased noradrenaline release enhance amygdala excitability (Davis, Rainnie, & Cassell, 1994). GABAergic inhibition by application of a GABA-A-receptor antagonist to the basolateral amygdala induced symptoms of anxiety in rats (Sanders & Shekhar, 1995). Accordingly, dysfunctional GABAergic inhibition was observed in patients with panic disorder (Ströhle et al., 2003). Binding at the GABA-A-receptor, benzodiazepines enhance GABAergic inhibition. Reduced binding sites for benzodiazepines have been demonstrated in patients with panic disorder and PTSD, indicating lower GABA transmission (Lydiard, 2003). This corresponds to a genotyping study revealing a linkage between panic disorder and specific candidate genes that define GABA-A-receptor characteristics (Hodges et al., 2014). Alternatively, reduced GABA transmission might result from increased noradrenaline levels in the amygdala, since noradrenaline suppresses GABAergic inhibition in the amygdala (Tully et al., 2007). Arousal evokes noradrenaline release from the LC (Tanaka, Yoshida, Emoto, & Ishii, 2000). Excessive noradrenaline release from the LC to the basolateral amygdala as it may occur during stressful experiences, might disrupt GABA transmission and thereby cause increased excitability of the amygdala due to increased glutamate transfer.

Beside this LC regulated circuit, the amygdala is also embedded in prefrontal regulatory circuits. Impairment of inhibitory frontal functions that support emotion regulation leads to elevated amygdala activation, also resulting in increased anxiety (Clausen et al., 2017; Linares et al., 2012; Shin & Liberzon, 2009). In the following, we review potential disruptions in those two major regulatory circuits in patients with anxiety disorders.

### The PFC-biomarker: disrupted prefrontal regulation in anxiety

Different regions of the PFC are involved in distinct aspects of emotional processing related to anxiety. An early model by Phillips (2003) suggested two distinct systems, a ventral and a dorsal one. Ventral regions of the anterior cingulate cortex (ACC) and PFC are involved in identification, evaluation of significance and production of affective states, while dorsal regions support effortful conscious emotion regulation (Phillips, Drevets, Rauch, & Lane, 2003). Patients with anxiety disorder show altered activation levels in both systems, however the direction of activation changes differs between studies.

Processing threat-related information has been associated with increased prefrontal activation in patients with anxiety disorders compared to healthy controls. When exposed to emotional stimuli, patients with PTSD revealed increased activation in the dorsolateral and ventromedial PFC (Bruce et al., 2013). Similarly, in patients with panic disorder, the anticipation of aversive sounds resulted in elevated activation in the amygdala, the ventromedial and ventrolateral PFC, as well as the dorsal ACC, dorsomedial and dorsolateral PFC (Brinkmann et al., 2017). In patients with generalised anxiety disorder, exposure to a narrative of threat-related content *v.* neutral content evoked increased activation in the ventrolateral and dorsomedial PFC, amygdala and thalamus relative to healthy controls, while activation in the ventromedial PFC and subgenual ACC was reduced relative to healthy controls (Buff et al., 2018). Social anxiety disorder was associated with increased activation in the amygdala, insula and the subgenual ACC in response to emotional faces (Ball et al., 2012; Labuschagne et al., 2012). Hence, the majority of studies reports elevated activation in ventral and dorsal portions of the PFC and ACC during perception and evaluation of emotional stimuli for patients with various types of anxiety disorders.

In healthy subjects, consciously up- and down-regulating emotions induce activation in the dorsal PFC and ACC while amygdala activation increases or decreases in accordance with the regulatory goal (Morawetz, Bode, Baudewig, & Heekeren, 2017). Reappraisal of a stimulus in order to downregulate a negative emotional response evokes increased activation levels in the dorsolateral PFC (Kohn et al., 2014; Ochsner & Gross, 2008; Stein, Simmons, Feinstein, & Paulus, 2007). A recent meta-analysis on fMRI studies that applied an emotional reappraisal task in patients with PTSD and various anxiety disorders concluded that these patients reveal decreased activation in the dorsomedial PFC and dorsal ACC in comparison with healthy controls when trying to regulate their emotions (Wang et al., 2018). As opposed to merely processing emotional cues, which is associated with increased ventral and dorsal PFC activation in patients with anxiety disorders, recruitment of dorsal route areas (dorsolateral PFC, supplementary motor area, dorsal ACC) in contexts that require emotion regulation is decreased in patients compared to healthy controls, resulting in increased anxiety.

Impaired PFC-amygdala communication is also reflected in altered functional PFC-amygdala connectivity. Patients with anxiety disorders have been characterised by reduced resting state connectivity between amygdala and dorsolateral PFC, and right amygdala and ventrolateral PFC, respectively (Jung et al., 2018; Makovac et al., 2016). In contrast, in healthy individuals, exposure to stress enhanced functional coupling between amygdala and dorsal ACC in a resting state period following moderate psychological stress (van Marle, Hermans, Qin, & Fernández, 2010). Negative coupling between the amygdala and dorsal ACC, pregenual ACC and posterior cingulate cortex related to the amount of perceived threat (van Wingen, Geuze, Vermetten, & Fernández, 2011). Hence, the appraisal of a potential threat affects amygdala connectivity and might alter amygdala regulation thereafter.

Taken together, increased amygdala sensitivity in anxiety disorders might either result from deficient PFC-based conscious regulatory processes, or stem from increased PFC excitability in response to the initial perception and evaluation of potentially threatening stimuli. Latter case might as well originate from elevation in noradrenaline transmission triggered by the LC during chronic phases of stress (Arnsten, 2009). High noradrenaline release to the PFC might potentiate overall excitability, intensifying the emotional affect and downregulate the executive control network which entails dorsal PFC regions involved in emotion regulation, while simultaneously increasing amygdala responsiveness (Hermans, Henckens, Joëls, & Fernández, 2014).

### The LC-biomarker: noradrenergic dysregulation in anxiety

Located in the pons, LC neurons are the major source of noradrenaline in the brain with monosynaptic connections throughout the entire central nervous system (Szabadi, 2013). Noradrenaline is a neuromodulator that shapes neural plasticity and firing properties of diverse neurons. Focusing on the amygdala as the most prominent anxiety-related structure, whole-cell recordings from amygdala slices demonstrated that noradrenaline release suppresses the inhibitory effect of GABAergic neurons and thereby increases the excitability of the amygdala (Tully et al., 2007). Accordingly, in the living rat, provoking increased noradrenaline transmission in the amygdala, hypothalamus and LC through administration of yohimbine, an *α*2-receptor antagonist, has been associated with anxiety symptoms in response to environmental stressors (Tanaka et al., 2000). This fear response might be specifically driven by noradrenergic projections from the LC to the amygdala. Earlier studies have shown that direct electrical or pharmacological stimulation of the LC in rats and mice induced fear (Bremner, Krystal, Southwick, & Charney, 1996) while bilateral lesion of the LC reduced fear (Neophytou, Aspley, Butler, Beckett, & Marsden, 2001). In humans, administration of the noradrenaline-reuptake-inhibitor reboxetine elevates activation in the basolateral amygdala in response to fearful faces (Onur et al., 2009), while the noradrenergic antagonist propranolol reduces basolateral amygdala activation in response to fearful faces (Hurlemann et al., 2010).

Interestingly, noradrenaline also affects a range of prefrontal processes. Selective activation of LC projections to the PFC in rats evokes anxiety-like behaviour and working memory impairment (Hirschberg, Li, Randall, Kremer, & Pickering, 2017). While medium rates of noradrenaline support attentional processes, a lack of noradrenaline leads to inefficiency and an excess of noradrenaline causes hypervigilance (Arnsten, 2009).

This non-linear effect following an inverted U-shape function might be based on the changing receptor preference of raising noradrenaline concentration. Blockade of *α*2-adrenergic receptors in the dorsolateral PFC leads to working memory deficits (Avery, Dutt, & Krichmar, 2013). While medium noradrenaline levels activate postsynaptic *α*2-adrenergic receptors and improve cognitive performance, high noradrenaline release stimulates postsynaptic *α*1-receptors in the PFC causing working memory impairment (Arnsten, 2000).

Taken together, increased noradrenaline release from LC neurons elevates amygdala excitability and weakens dorsolateral PFC functions (Arnsten et al., 2015). Hence, top-down control onto the amygdala might be disrupted while amygdala sensitivity is further strengthened via noradrenergic modulation of GABAergic processes.

Anxiety symptoms in psychiatric patients are associated with overall increased noradrenaline transmission (Yamamoto, Shinba, & Yoshii, 2014). Patients with PTSD reveal elevated baseline levels of noradrenaline in the cerebrospinal fluid and exposure to trauma-related cues increase these levels even further (Geracioti et al., 2001; Strawn & Geracioti, 2008). It remains unclear if pre-traumatic elevated noradrenaline levels are causal in the vulnerability to develop PTSD or if elevated noradrenaline transmission results from the psychopathological state itself. Yet, imbalances in noradrenaline might stem from genetic predispositions. Panic disorder and increased anxiety traits were associated with reduced expression of SLC6A2, which regulates noradrenaline homeostasis (Hommers et al., 2018).

The *α*2-adrenergic receptor agonist clonidine diminishes noradrenaline release by activating inhibitory autoreceptors at presynaptic sites in the LC (Olson et al., 2011). Major therapeutic effects of clonidine are attained in treatment of hypertension, and during acute opioid withdrawal where it blocks elevated startle response (Gregoretti, Moglia, Pelosi, & Navalesi, 2009). Fewer binding sites for clonidine in patients with anxiety disorders indicate that a decreased amount of inhibitory autoreceptors might result in elevated noradrenaline levels and provoke anxiety (Cameron et al., 1990). Therapeutic effects of clonidine have been reported for symptoms like hyperarousal, hypervigilance, sleep disruption and re-enactment (Arnsten et al., 2015; Detweiler et al., 2016). Hence, sensitisation of the noradrenergic system might contribute to arousal symptoms associated with anxiety (Arnsten et al., 2015). However, in other studies clonidine infusion did not lead to symptom reduction (Hood et al., 2011; Kalk et al., 2012). Furthermore, the administration of the *α*2-receptor agonist guanfacine, which also activates presynaptic *α*2-receptors did not cause effective symptom reduction (Neylan et al., 2006). Variable results and differences in drug effects might stem from variation in the drug or might be based on heterogeneity in terms of underlying pathophysiology.

While *α*2-receptors are presynaptically involved in a negative feedback loop, downregulating noradrenaline release, *α*1-receptors are located postsynaptically. Anxiety-related symptoms have been treated successfully with prazosin, an *α*1-adrenergic receptor antagonist that blocks postsynaptic *α*1-receptors and reduces trauma-related nightmares and overall symptom severity (Keeshin, Ding, Presson, Berkowitz, & Strawn, 2017; Peskind, Bonner, Hoff, & Raskind, 2003; Raskind et al., 2013; Taylor et al., 2006). However, drugs acting on neurotransmitter systems are not entirely selective for one receptor type. More trials are needed to clarify the therapeutic effect of manipulating noradrenaline transmission by targeting adrenergic receptors, also in combination with the CBT-based state of the art treatments.

Increased noradrenaline levels might reduce benefits from CBT. Increased pupil dilation in response to emotional faces was related to reduced treatment benefit from CBT in patients with social anxiety disorder (Kleberg, Hanqvist, Serlachius, & Högström, 2019). As noradrenaline is associated with pupil dilation, these findings might indirectly point to underlying imbalances in the noradrenaline system dampening the effect of CBT.

Considering the prominent role of noradrenaline in vigilance processes, it might be concluded that agents acting on the noradrenaline system lead to the relief of a wide array of anxiety symptoms. Due to limited evidence, treatment of anxiety with agents interacting with noradrenergic receptors is no common clinical practice. Limited efficacy of agents acting on noradrenaline might be based on a missing general effect, since patients might differ regarding their pathophysiological mechanism. In the broader framework of this model, divergent findings might point out the overall heterogeneity of underlying pathology among patients.

### The 3-biomarker model

**Fig. 1.**
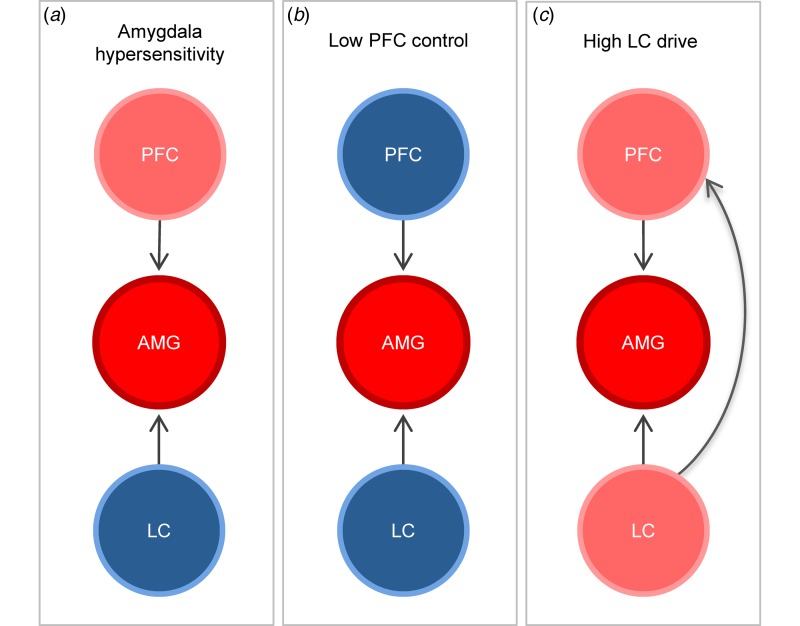
(*a*) Despite high PFC control, increased amygdala (AMG) response occurs due to inherent amygdala hypersensitivity. (*b*) A lack of emotion regulation is based on low PFC control, which results in increased amygdala responsiveness. (*c*) Elevated noradrenaline release due to high LC drive leads to increased amygdala responsiveness and distraction in cortical processes involved in emotion processing.

Taken together, we suggest that the biological origin of amygdala hyper-responsiveness might be based on imbalances in amygdala-centred regulatory mechanisms, consisting of three core regulatory hubs (biomarkers) that might upregulate amygdala responsiveness. The amygdala-biomarker is defined by disruption of GABA-based inhibitory processes within the amygdala. The PFC-biomarker is characterised by reduced recruitment of inhibitory top-down projections. The LC-biomarker is based on increased noradrenaline release from the LC, which drives up amygdala activation by disrupting GABAergic inhibition and intensifies emotion processing by amplifying attentional processes. Yet, those three biomarkers form an amygdala-centred network and manifestations in one biomarker might affect the other mechanisms driving the network (Fig. 1).

## Finding the right match: biomarker guided treatment of anxiety

We propose inherent amygdala hypersensitivity or alterations in amygdala-centred regulatory circuits (reduced dorsal PFC recruitment during emotion regulation and increased LC-drive during stress) all lead to the same fearful phenotype. Although the three mechanisms might be interconnected, and targeting one mechanism would affect amygdala responsiveness either way, we propose that individualised treatment selection that specifically targets the causative neural biomarker in a given patient would increase treatment efficiency. Targeting the causative biomarker would treat an anxiety disorder at its core mechanism and would allow patients to be allocated to a treatment according to the biological origin of their anxiety. This might reduce expensive and time-consuming detours in treatment procedures. Importantly, this section aims to give an outlook on the potential impact of biomarker characterisation on clinical practise. Deriving treatment guidelines from biomarkers is still a matter of research and this work intends to stimulate future studies on biomarkers with a focus on treatment implications.

### Targeting amygdala hypersensitivity

In this model, we assume that patients with amygdala hypersensitivity experience symptoms of anxiety despite being capable of appropriate cognitive emotion regulation strategies. This might indicate that CBT does not target amygdala hypersensitivity. As discussed above, disruption of GABAergic inhibition might be the underlying neurobiological mechanism of amygdala hypersensitivity. To specifically target disruption of GABAergic inhibition, treatment with benzodiazepines might be indicated. Benzodiazepines enhance GABAergic inhibition and have been demonstrated as effective treatment, preferably as short-term treatment for patients with anxiety symptoms that have not responded to other treatments (Baldwin et al., 2005).

Considering the burden of side effects and issues of sustained success, psychotherapeutic treatment might be an alternative. Yet, it remains unclear if any psychotherapeutic treatment is less effective in patients with amygdala hypersensitivity as compared to patients having impaired prefrontal control. Potentially, patients with predominant amygdala hypersensitivity might show more physiological symptoms of anxiety and those tend to become the object of fear. Interoceptive exposure to reappraise physiological symptoms has been demonstrated to efficiently diminish symptoms of anxiety (Holtz, Hamm, & Pané-Farré, 2019). Similarly, mindfulness-based interventions train a detached observational state that might attenuate negative emotions and have been demonstrated to reach clinical improvement in patients with anxiety disorders (Goldin & Gross, 2010). On the neural level, mindfulness training has been shown to decrease amygdala responses during emotional processing (Taylor et al., 2011).

### Prefrontal engagement in emotion processing *v.* emotion regulation predicts exposure-based CBT outcome

Within the framework of the proposed model, a lack of prefrontal recruitment during emotion regulation is classified as a PFC-biomarker. We assume that patients with a PFC-biomarker have a higher benefit from exposure-based CBT than patients with a different biomarker, since exposure-based CBT specifically increases PFC recruitment during emotion regulation (Goldin et al., 2013).

So far, therapy outcome has not been evaluated after stratification of patients based on different biomarkers prior to treatment. Yet, studies report *post-hoc* predictions of treatment benefit based on PFC recruitment during emotion regulation and emotion perception before exposure-based CBT.

In patients with social anxiety disorder, reduced dorsolateral PFC activation during emotional reappraisal prior to treatment predicted better treatment outcome of CBT (Klumpp et al., 2017a). Also in a sample of patients with various anxiety disorders and major depression, reduced ACC response to an emotion regulation task and increased ACC activation during a threat interference paradigm defined treatment responders (Klumpp et al., 2017b). The underlying cognitive process triggered by the experimental paradigm might define the direction of activation predicting the treatment response. Hence, decreased activation during emotion regulation and increased activation during emotional processing were associated with the better treatment response. Sorting studies based on the experimental paradigm, it appears that studies that applied paradigms that tap into emotional processing without the attempt of emotion regulation report increased PFC recruitment as a predictor for treatment response. Patients with PTSD that initially showed greater engagement of dorsolateral PFC, dorsal ACC and left amygdala during an emotional reactivity task, experienced more symptom reduction after exposure therapy, while greater baseline activation of the ventromedial PFC as well as ventral striatal activation during an emotional conflict task was associated with better treatment outcome from exposure therapy (Fonzo et al., 2017). Similarly, in patients with social anxiety disorder, greater activation of the pregenual ACC, ventromedial PFC/middle frontal gyrus and left amygdala in response to social rejection cues were associated with higher symptom reduction after exposure (Burklund, Torre, Lieberman, Taylor, & Craske, 2017). Prior enhanced dorsal ACC and dorsomedial PFC activation in response to emotional faces were also associated with better outcome from exposure therapy in social anxiety disorder (Klumpp, Fitzgerald, Angstadt, Post, & Phan, 2014).

Hence, initially increased prefrontal activation during emotional processing might be associated with a more salient discrimination between threatening and non-threatening stimuli, which facilitates symptom reduction during exposure therapy. Activation of the dorsal ACC and the amygdala predicted treatment outcome of internet-based CBT with an accuracy of 92% (Mansson et al., 2015). Moreover, patients with PTSD that revealed enhanced amygdala and right ventral ACC activation during non-conscious threat processing showed a poor treatment response, indicating that the excessive amygdala response might as well hinder treatment success (Bryant et al., 2008). Despite heterogeneous results, a systematic review on biomarkers predicting the treatment outcome in anxiety disorders reports increased dorsal ACC and ACC-amygdala interaction related to threat processing as a predictor for the exposure-treatment response (Lueken et al., 2016).

Overall, two lines of argumentation can be inferred. Initially decreased prefrontal recruitment during regulatory processes might predict higher treatment benefit, since decreased prefrontal engagement can be balanced by exposure-based CBT where regulatory strategies are trained. Exposure-based CBT directly affects prefrontal activation in patients with social anxiety disorder, also increased dorsolateral as well as dorsomedial PFC activation, and strengthened inverse dorsomedial PFC-amygdala connectivity during an emotion regulation task (Goldin et al., 2013). Furthermore, exposure treatment led to enhanced amygdala-ventromedial PFC connectivity during an affect-labelling task, while symptom reduction was associated with more negative amygdala-ventromedial PFC connectivity (Young et al., 2017). Hence, exposure therapy might balance prior deficiencies in regulatory areas. In contrast, elevated activation during emotion processing before treatment might as well relate to treatment response. Patients who initially recruit prefrontal resources for emotional processing seem to benefit from a therapy form that reinforces the distinction between threat-related and safe stimuli.

The precise characterisation of a PFC-biomarker remains challenging. More refined localisation of cortical regions is restricted by heterogeneous functional labels of anatomical locations across studies. A precise distinction between the triggered cognitive processes might be the first step towards clarification. Studies applied various paradigms that either trigger emotion perception, conscious emotion regulation or presumably tap into both processes. Furthermore, model-based approaches restricting the analysis to predefined regions of interest eventually lead to different results than if predictive regions are derived from whole brain analyses. The diverse outcome might as well reflect neurobiological heterogeneity among patients. Study populations have been too restricted in size to identify predictive effects due to potential heterogeneity of underlying biomarkers. Hence, studies that report increased PFC activation in patients with anxiety disorders might either tap into emotion processing instead of regulatory processes, or indicate a different underlying biomarker. Within the framework of the proposed biomarker model we suggest that increased PFC activation during emotion processing might stem from increased noradrenaline release from the LC to prefrontal regions in response to threat. Increased noradrenaline influx to the PFC would elevate PFC activation. While intermediate levels of noradrenaline allow optimal PFC control, increased noradrenaline levels harm PFC efficiency by exceeding the optimal activation level (Arnsten, 2009). In the case of increased noradrenaline levels being the primary cause of elevated PFC activation, treatment might be supported by targeting the noradrenergic system. Hence, a patient with increased PFC recruitment during threat should be tested for increased LC-drive in order to eventually increase treatment efficiency by administration of noradrenaline antagonists.

### Treatment of noradrenergic hyperfunction

**Fig. 2.**
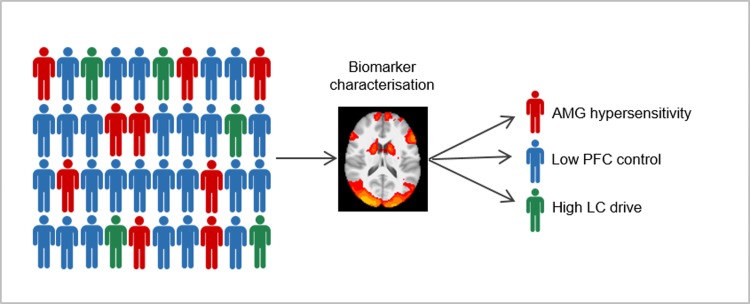
Biomarker characterisation in patients with anxiety disorders based on the three potential mechanisms of anxiety: amygdala hypersensitivity, low PFC control and high LC drive may provide a heuristic for pathology-guided treatment selection. Inherent amygdala hypersensitivity indicates treatment with GABA-based medications like benzodiazepines, low PFC control can be treated by exposure interventions, and high LC drive might be targeted by noradrenergic agents.

We suggest increased LC drive as another biomarker of anxiety disorders. Controlling for altered noradrenergic release from the LC might reveal if increased PFC activation originates from increased LC drive, and thus would be treated more effectively by targeting noradrenergic hyperfunction (Fig. 2).

In monkeys, increased tonic LC activation disrupts prefrontal processes related to attention as it led to weaker performance in a discrimination task (Aston-Jones, Rajkowski, & Cohen, 1999). This might implicate that in humans, the evaluation of an emotional cue might be disturbed by increased tonic LC activation. Also, increased LC activation induces physiological symptoms of stress, which might bias the evaluation of the outside world as being more threatening. Patch-clamp recordings in rats have shown that LC cells that are projecting to the medial PFC have a lower excitation threshold than LC neurons projecting to other cortical areas, like the motor cortex (Chandler, Gao, & Waterhouse, 2014). Thus, increased LC activation might have a stronger impact on cognitive processes that involve the medial PFC. Transferring these findings to emotion processing in humans, high LC drive might maintain increased PFC activation during emotion processing making effective emotion regulation more difficult. Emotion regulation is trained in exposure-based CBT and hence, high LC drive might interfere and reduce treatment effects. Although increased noradrenaline transmission (indirectly measured via noradrenaline concentration in CSF samples) has been reported for patients with PTSD (Geracioti et al., 2001; Strawn & Geracioti, 2008), and effective treatment with noradrenaline antagonists has been demonstrated (Raskind et al., 2013; Taylor et al., 2006), there are to our knowledge no studies investigating the predictive value of measuring initial noradrenaline levels on treatment outcome of exposure therapy, or pharmacological approaches that target the noradrenergic system.

Considering the prominent effect of noradrenaline on physiological symptoms of anxiety, psychotherapeutic approaches that rather focus on physiological symptoms and cognitive distraction like attention deficits and working memory impairment might be a more sustainable alternative for patients with increased LC drive. Tonic firing of the LC is present during high arousal, and associated with mind-wandering. Mindfulness-based programs train attention, reduce mind-wandering and might decrease tonic LC-firing (Russell & Arcuri, 2015). However, a potential direct effect of mindfulness techniques on noradrenaline release has not been investigated yet. Prospective studies are necessary to investigate the potential link between noradrenaline and mind-wandering.

## Outlook

### Biomarker characterisation based on neuroimaging data

**Fig. 3.**
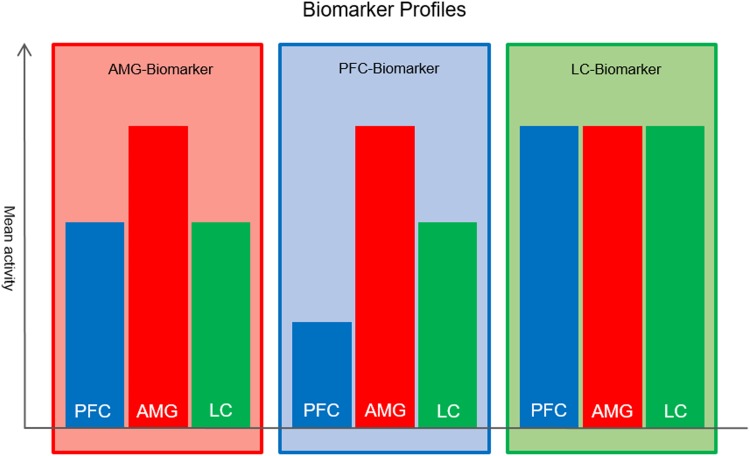
Based on the model, all three biomarkers reveal increased amygdala (AMG) activation. For patients with amygdala hypersensitivity (AMG biomarker) increased AMG activation is the key feature, while PFC and LC activation are not deviating in this biomarker. The PFC biomarker is characterised by low PFC activation. Patients with an LC biomarker would express increased LC activation as a key feature, while PFC activation might increase due to regulatory attempts of the PFC. Yet, high noradrenaline release from the LC to the PFC might upregulate PFC activation, causing to exceed the optimal activation level of the PFC.

Specifying predictive biomarkers require large cohort studies that allow stratification of patients based on the activity of their anxiety evoking neural mechanism. We propose an fMRI-based study design combining and integrating three behavioural tasks that trigger activation in the three regions of interest. A supervised classification and clustering approach based on activity within and the dynamic interaction between the core hubs of the amygdala-centred regulatory mechanisms might be used to stratify a highly anxious population into three mechanistically defined clusters of individuals (a) amygdala sensitivity, (b) low PFC control and (c) high LC drive. An emotion regulation task could be applied to monitor activation in the PFC during regulatory attempts (Ochsner, Bunge, Gross, & Gabrieli, 2002). Amygdala reactivity could be monitored by applying a face recognition task (Hariri, Bookheimer, & Mazziotta, 2000). Salience processing (i.e. an oddball task) in combination with a threat-of-shock paradigm could monitor LC activation (Hermans et al., 2011). Manipulation of alertness by applying a threat-of-shock paradigm would indirectly manipulate the level of phasic LC activation, while an oddball task would trigger tonic activation. This approach would allow defining a neural profile for each biomarker. Based on the correspondence of the neural profile of a patient, the primary biomarker could then be determined (Fig. 3).

Alternatively, stratification on functional connectivity data during resting state scans might be another avenue to take. A PFC biomarker would be characterised by reduced resting state connectivity between amygdala and dorsolateral PFC, and right amygdala and ventrolateral PFC, respectively (Jung et al., 2018; Makovac et al., 2016). LC activation could be measured by a neuromelanin scan (Sasaki et al., 2006), and the amygdala biomarker might be present in cases that neither reveal reduced amygdala-PFC connectivity nor increased LC activation.

A selected array of physiological measures that are known to be related to the three regions of interest might help to distinct the three biomarkers. Pupil dilation is a reliable proxy for LC hyperactivity (Gilzenrat, Nieuwenhuis, Jepma, & Cohen, 2010). Heart rate has been associated with increased prefrontal activation and efficacy of emotion regulation processes (Makovac, Thayer, & Ottaviani, 2017; Thayer & Lane, 2000). Characterising patients along additional physiological measures might be of advantage, as secondary effects of a biomarker mechanism on the other two mechanisms would be potentially less pronounced on the secondary physiological readouts.

### Predictive biomarkers in clinical practice

Defining a biomarker in individual patients might allow personalised therapy selection tailored to the individual neural disposition of a given patient. Studies are needed that evaluate therapy induced activation changes during emotion processing and emotion regulation separately and evaluate, if exposure-based CBT balances initial alterations of activation during those processes. The predictive value of prefrontal control regarding treatment success of exposure therapy might be clarified by considering noradrenaline transmission as a second factor. Increased noradrenaline release to the PFC, specifically to regions underlying emotion processing, might prevent therapy-induced reduction of initially increased activation levels and therefore predict reduced benefit from CBT. Thus, in a case of increased noradrenaline (LC biomarker) the model rather supports an intervention that downregulates LC drive. However, it is unknown if decreased PFC activation causes increased noradrenaline levels. The existence of a potential association between decreased PFC control and increased noradrenaline levels would need to be clarified in order to establish a reliable noradrenaline-based predictor for the CBT outcome. Furthermore, the LC might not be a homogenous modulator of PFC activation but might have a stronger effect on medial as compared to other frontal areas (Chandler et al., 2014).

## Limitations

The proposed model is highly simplified and only considers three biomarkers as the origin of increased amygdala response. Animal studies attribute a crucial role to dopamine and serotonin regarding extinction learning, which potentially might have predictive value as well (Singewald, Schmuckermair, Whittle, Holmes, & Ressler, 2015). Furthermore, this model is focussed on neural mechanistic interactions of three neural mechanisms. It is to be expected that a combination of genetic and environmental factors are causing these deficits, but the exact contributions of genetic variance and factors like experience and learning history are currently unknown. Proposed treatment indications are based on the assumption that targeting the causative biomarker might be more efficient. There is no direct evidence yet that biomarker-targeted treatment would increase treatment benefit in the context at issue. To provide a heuristic model for treatment selection, measurements need to be clinically feasible and thus, individualised. A neuroimaging-based biomarker characterisation might not be clinically feasible, however reliable indirect physiological measurements as proxies for neural mechanisms as well as reference frames to categorise patients based on those are still a matter of research.
